# RETRACTION: F806 Suppresses the Invasion and Metastasis of Esophageal Squamous Cell Carcinoma Via Downregulating F‐Actin Assembly‐Related Rho Family Proteins

**DOI:** 10.1155/bmri/9831425

**Published:** 2026-08-12

**Authors:** BioMed Research International

RETRACTION: L. Xie, L.‐Y. Li, D. Zheng, Y.‐M. Xie, X.‐E. Xu, L.‐H. Tao, L.‐D. Liao, Y.‐H. Xie, Y.‐W. Cheng, L.‐Y. Xu, and E.‐M. Li, “F806 Suppresses the Invasion and Metastasis of Esophageal Squamous Cell Carcinoma via Downregulating F‐Actin Assembly‐Related Rho Family Proteins,” *BioMed Research International*, 2018, (2018): 2049313, https://doi.org/10.1155/2018/2049313

The above article, published online on 19 September 2018 in Wiley Online Library (wileyonlinelibrary.com), has been retracted by agreement between journal Associate Editor, Jinsong Ren; and John Wiley & Sons Ltd. The retraction has been agreed due to figure concerns that have been raised on PubPeer [1]. Specifically:

• In the EC109 images of Figure 4d, the control Paxillin panel appears to be a magnified duplicate of the EC109 control panel in Figure 4a of [2]
• In the KYSE510 images of Figure 4d, the control Paxillin panel appears to be a duplicate of the KYSE510 FN control panel in Figure 4a of [2]

The authors did provide original data when informed of the retraction, however after assessment by the editorial board the results and conclusions can no longer be considered reliable, and the article is retracted.

The authors did not respond when asked to indicate their agreement to the retraction.
